# Conformational Equilibrium of NADPH–Cytochrome P450 Oxidoreductase Is Essential for Heme Oxygenase Reaction

**DOI:** 10.3390/antiox9080673

**Published:** 2020-07-28

**Authors:** Masakazu Sugishima, Junichi Taira, Tatsuya Sagara, Ryota Nakao, Hideaki Sato, Masato Noguchi, Keiichi Fukuyama, Ken Yamamoto, Takuo Yasunaga, Hiroshi Sakamoto

**Affiliations:** 1 Department of Medical Biochemistry, Kurume University School of Medicine, 67 Asahi-machi, Kurume 830-0011, Japan; hsato@med.kurume-u.ac.jp (H.S.); miwata2410@384.jp (M.N.); yamamoto_ken@med.kurume-u.ac.jp (K.Y.); 2 Department of Bioscience and Bioinformatics, Graduate School of Computer Science and Systems Engineering, Kyushu Institute of Technology, 680-4 Kawazu, Iizuka 820-8502, Japan; taira@bio.kyutech.ac.jp (J.T.); supersonic.runrun@gmail.com (T.S.); nakryo@gmail.com (R.N.); yasunaga@bio.kyutech.ac.jp (T.Y.); 3 Department of Biological Sciences, Graduate School of Science, Osaka University, 1-1 Machikaneyama-cho, Toyonaka 560-0043, Japan; fukuyamakei1@gmail.com

**Keywords:** analytical ultracentrifuge, cryo-electron microscopy, electron transfer, protein–protein interaction

## Abstract

Heme oxygenase (HO) catalyzes heme degradation using electrons supplied by NADPH–cytochrome P450 oxidoreductase (CPR). Electrons from NADPH flow first to FAD, then to FMN, and finally to the heme in the redox partner. Previous biophysical analyses suggest the presence of a dynamic equilibrium between the open and the closed forms of CPR. We previously demonstrated that the open-form stabilized CPR (ΔTGEE) is tightly bound to heme–HO-1, whereas the reduction in heme–HO-1 coupled with ΔTGEE is considerably slow because the distance between FAD and FMN in ΔTGEE is inappropriate for electron transfer from FAD to FMN. Here, we characterized the enzymatic activity and the reduction kinetics of HO-1 using the closed-form stabilized CPR (147CC514). Additionally, we analyzed the interaction between 147CC514 and heme–HO-1 by analytical ultracentrifugation. The results indicate that the interaction between 147CC514 and heme–HO-1 is considerably weak, and the enzymatic activity of 147CC514 is markedly weaker than that of CPR. Further, using cryo-electron microscopy, we confirmed that the crystal structure of ΔTGEE in complex with heme–HO-1 is similar to the relatively low-resolution structure of CPR complexed with heme–HO-1 in solution. We conclude that the “open–close” transition of CPR is indispensable for electron transfer from CPR to heme–HO-1.

## 1. Introduction

Heme oxygenase (HO, EC 1.14.14.18) catalyzes the degradation of heme to biliverdin, CO, and ferrous ion [1,2,3] by utilizing reducing equivalents derived from NADPH–cytochrome P450 reductase (CPR, EC 1.6.2.4). The major physiological roles of an inducible isoform of HO, HO-1, in mammals are the maintenance of iron homeostasis by the recycling of iron and the defense against oxidative stress by the detoxification of heme, a pro-oxidant, and the production of bilirubin, a potent antioxidant. CO, produced by HO-1 and a constitutive isoform, HO-2, mediates various types of cell activities, such as anti-inflammatory, anti-apoptotic, and vasodilatory activities [4,5]. The HO reaction proceeds via three reaction intermediates: α-hydroxyheme, α-verdoheme, and biliverdin–iron chelate. CO is produced in the conversion from α-hydroxyheme to α-verdoheme. At least three of four reaction steps, reducing equivalents supplied by CPR, are required. In total, seven electrons are required for a cycle of reaction to produce biliverdin and ferrous ion from hemin. Biliverdin is subsequently converted to bilirubin by biliverdin reductase [6,7,8]. Almost all crystal structures of HO complexed with its substrates, reaction intermediates, and product have been determined [9,10,11,12,13,14,15,16,17,18,19].

CPR is a member of a family of diflavin reductases, which catalyzes electron transfer from NADPH to FAD, FMN, and finally the heme groups in their redox partners [20,21]. Rat CPR (rCPR) is composed of three domains: the FMN-binding domain (residues 77–232), the ferredoxin–NADP^+^ oxidoreductase (FNR)-like domain (residues 267–325 and 450–678), and the connecting domain (residues 244–266 and 326–450). The connecting domain and the FNR-like domain jointly form the FAD-binding domain, wherein the NADPH-binding site is present. The FAD and FMN-binding domains are connected by a flexible hinge formed by a span of 12 residues, from Gly232 to Arg243, in rCPR.

For electron transfer to occur, CPR and its redox partners must associate with one another. The first reported CPR structure was observed to have a closed conformation, in which NADP^+^, FAD, and FMN were in close proximity, which was suitable for intramolecular electron transfer [22]. However, it is hardly probable that redox partners such as cytochrome P450 could be positioned close enough to FMN for intermolecular electron transfer to occur.

Hamdane et al. developed an rCPR mutant in which four consecutive residues in the hinge region (from Thr236 to Glu239) were removed (hereafter referred to as ΔTGEE). They determined the crystal structure of ΔTGEE and observed three remarkably extended conformations (open conformation). In the open conformation, the distance between FAD and FMN coenzymes were in the range of 30–60 Å [23]. The structures of ΔTGEE indicate that the FMN-binding domain is highly mobile compared to the rest of the molecule, whereby the open conformation of CPR is able to bind its redox partner. Recently, we were able to determine the crystal structure of ΔTGEE complexed with heme–rat HO-1 (rHO-1) [24] and observed that ΔTGEE could bind its redox partner tightly and change its conformation marginally upon NADP^+^/NADPH binding [25]. The results of the NMR analysis of HO-2 complexed with CPR [26] are consistent with the crystallographic results. However, the distance between FAD and FMN in the complex is considerably large to allow direct electron transfer, and the reduction kinetics of heme–HO-1 in the presence of the NADPH–ΔTGEE system is 360-fold slower than of that in the presence of the NADPH–rCPR system. Moreover, Xia et al. developed an rCPR mutant in which the salt bridge (Asp147–Arg514) between the FMN-binding and the FAD-binding domains in the closed conformation was replaced by a disulfide bridge to stabilize the closed conformation (hereafter referred to as 147CC514) [27]. Intramolecular electron transfer in 147CC514 was impaired by the rotation of FMN by 20° and the weakened affinity of NADPH, while the rate of hydride transfer from NADPH to FAD was moderately reduced. Intermolecular electron transfer to cytochrome *c* and cytochrome P450 was severely impaired by 147CC514. Further, it was reported that these transfers were restored upon cleavage of the disulfide bond in 147CC514 in response to the addition of dithiothreitol (DTT). These data suggest that the “open–close” transition of CPR is necessary for a smooth electron transfer. Computational simulation provided evidence of the “open–close” transition of CPR with the redox change in its coenzymes [28].

However, to complete a catalytic cycle, HO utilizes seven electrons supplied by CPR, whereas cytochrome P450 utilizes only two electrons. Since electron transfer from CPR to heme–HO-1 occurs sequentially, seven cycles of “open–close” transition of CPR are required for a single cycle of HO reaction. During the HO reaction, oxygen-labile reaction intermediates such as α-hydroxyheme and α-verdoheme are formed. Therefore, if the electron transfer cycle from CPR to HO is considerably slow, these intermediates may be decomposed before they can accept the next electron. However, such decomposition does not occur under physiological conditions. To validate the current electron transfer model, we characterized the HO reaction and the reduction kinetics of heme–rHO-1 in the presence of the NADPH–147CC514 system. Further, we characterized the interaction of heme–rHO-1 with rCPR, ΔTGEE, and 147CC514 by analytical ultracentrifugation. In addition, we confirmed that the structure of rCPR complexed with heme–rHO-1 in solution is similar to the crystal structure of the ΔTGEE–heme–HO-1 complex, using single-particle analysis in cryo-electron microscopy in spite of its low resolution. On the basis of these results, we confirmed that the “open–close” transition of CPR is necessary for a smooth electron transfer from CPR to heme–HO-1.

## 2. Materials and Methods

### 2.1. Protein Expression and Purification

Both rCPR and rHO-1 are membrane-bound proteins anchored to the cytoplasmic surface of the endoplasmic reticulum. To handle these conveniently, we prepared soluble forms of rHO-1, rCPR, ΔTGEE, and 147CC514 by removing their membrane-spanning regions. rHO-1, rCPR, and ΔTGEE were expressed in *Escherichia coli* and purified as described earlier [23,24,29,30]. An expression vector was constructed for 147CC514 using the synthesized cDNA (FASMAC, Atsugi, Japan) of rCPR in which Asp147 and Arg514 were substituted with cysteine residues, and except for the catalytic cysteine (Cys630), the other cysteine residues in rCPR not containing the membrane-spanning region (Met1-Ile57), were substituted with amino acid residues to form Cys136Ala, Cys228Ala, Cys363Thr, Cys445Leu, and Cys472Thr [27]. The resultant cDNA of 147CC514 was subcloned into a pET-21a(+) expression vector (Merck, Darmstadt, Germany) for the expression of the non-His tagged enzyme, using an in-fusion HD cloning kit (Takara Bio, Kusatsu, Japan) and the appropriate primers. The pET-21a-147CC514 plasmid formed, in which the open reading frame (ORF) sequence was confirmed by DNA sequencing, was transformed into Rosettagami B (DE3) (Merck), followed by overnight culturing of the transformed *E. coli* at 37 °C on Luria–Bertani (LB)–agar plates containing 100 μg/mL ampicillin and 33 μg/mL chloramphenicol. A single colony was selected to inoculate 3 mL of LB medium containing 100 μg/mL ampicillin and 33 μg/mL chloramphenicol; 1.6 mL of the overnight culture was used to inoculate 400 mL of LB medium containing 100 μg/mL ampicillin, 33 μg/mL chloramphenicol, and 10 μg/mL riboflavin. The cells were cultured in baffled flasks at 37 °C with shaking at 240 rpm for 3 h, at which point isopropyl β-D-1-thiogalactopyranoside (IPTG) was added to a final concentration of 100 μM to initiate reductase expression. The cultures were incubated for an additional 72 h at 19 °C with shaking at 130 rpm.

The cells expressing 147CC514 were sonicated in a solution with 50 mM Tris/HCl (pH 8.0), 2 mM EDTA (pH 8.0), 20% (v/v) glycerol, and 0.1% (v/v) Triton X-100. The mixture was incubated on ice for 3 h. Next, the insoluble fraction was removed by centrifugation. The 147CC514 protein was purified by the method used for rCPR purification [30], using anion-exchange, affinity, and hydroxyapatite columns with slight modifications (addition of 0.1% Triton X-100). Briefly, the supernatant was loaded in a Hitrap Q HP column (GE Healthcare, Chicago, IL, USA) equilibrated with 20 mM Tris/HCl (pH 7.4), 0.1 mM EDTA, 20% glycerol, and 0.1% Triton X-100. The yellow fractions eluted with a linear gradient of potassium chloride were collected. Subsequently, the collected fractions were introduced into a 2′,5′-ADP Sepharose column (GE Healthcare) equilibrated with 5 mM potassium phosphate (pH 7.7), 20% glycerol, and 0.1% Triton X-100. After washing with the equilibration buffer and adding 0.7 mM NAD^+^, the 147CC514 fraction was eluted using the equilibration buffer, adding 0.7 mM NADP^+^. Excess NADP^+^ was removed using a Bio-Scale Mini CHT Type I Cartridge (Bio-Rad, Hercules, CA, USA) equilibrated with 5 mM potassium phosphate (pH 7.7) and 20% glycerol. The formation of the disulfide bond between Cys147 and Cys514 was confirmed by X-ray crystallography (Figure S1A, Table S1). The crystallization conditions were the same as reported by Xia et al. [27]. Diffraction data were merged and scaled with XDS package [31]. The model (PDB ID: 3OJW) was refined with Refmac5 [32] in CCP4 package [33]. The overall structure appeared to be almost identical to that reported by Xia et al. [27], although some loops (239–241, 501–504, and 598–600) were disordered in our refined model (Figure S1B). NADP^+^ was bound to the crystal, whereas the electron density of the adenine ring of NADP^+^ was low. The electron density of the nicotinamide ring of NADP^+^ and that of rCPR were low as well [22].

DTT-treated and 2-iodoaceteamide (IAM)/DTT-treated 147CC514 were prepared according to the method reported by Xia et al. [27]. Briefly, DTT-treated 147CC514 was prepared by incubating 147CC514 with DTT for 40 h at 4 °C in the UNIlab Pro Glove Box (MBRAUN, Garching bei München, Germany), following which, the excess DTT was removed using a Zeba desalt spin column (Thermo Fisher Scientific, Waltham, MA, USA). Subsequently, IAM/DTT-treated 147CC514 was prepared by incubation of the DTT-treated 147CC514 with IAM for 30 min at 25 °C in an anaerobic chamber, following which, excess IAM was removed using a Zeba desalt spin column. To completely remove DTT or IAM, the samples were desalted twice in each step. For the control experiments, DTT-treated rCPR and IAM/DTT-treated rCPR were prepared according to the method used for 147CC514 preparation. The concentrations of rCPR, ΔTGEE, and 147CC514 were determined by measuring the absorbance at 454 nm with a molar extinction coefficient (ε) of 21.4 mM^−1^ cm^−1^.

The heme–rHO-1 complex was reconstituted with 1.2 equiv. of heme and purified by column chromatography on a hydroxyapatite column (Bio-Rad), as previously described [34]. The concentration of heme–rHO-1 was determined by measuring the absorbance at 406 nm (ε = 140 mM^−1^ cm^−1^) (pH 7.4). Rat biliverdin reductase used for the enzymatic assay was expressed in *E. coli* and purified by ammonium sulfate fractionation, affinity chromatography (2′,5′-ADP Sepharose), and size-exclusion chromatography (Sephacryl S-200 HR (GE Healthcare)) [7,35].

### 2.2. Enzymatic Assay

HO activity in the NADPH–CPR system was determined based on the rate of bilirubin formation, which was monitored by the increase in the absorbance at 468 nm at 37 °C [36]. Bilirubin formation from biliverdin was catalyzed by biliverdin reductase present in the assay mixture, which contained 40 μM heme, 0.5 μM rHO-1, 0.5–2 μM rCPR—or 4–12 μM 147CC514, 1–1.5 μM IAM/DTT-treated rCPR, or 1–2 μM IAM/DTT-treated 147CC514—0.5 mg/mL bovine serum albumin, 12.5 μg/mL catalase, 6.7 μg/mL biliverdin reductase, and 250 µM NADPH in 0.1 M potassium phosphate buffer (pH 7.4). The assay commenced upon the addition of NADPH. A typical example of the assay is shown in Figure S2A.

Single turnover reactions were monitored based on changes in the absorption spectra at 30 °C. The reaction mixtures (0.1 mL) consisted of 4.3 µM heme–rHO-1, 40 nM rCPR or 100 nM 147CC514, and 25 µM NADPH in 0.1 M potassium phosphate buffer (pH 7.4). The spectra were recorded over a range of 300–900 nm.

Heme reduction was monitored based on changes in the absorption spectra at 30 °C in a CO-saturated anaerobic atmosphere. The CO-saturated reaction mixtures (0.1 mL) consisted of 4.3 µM heme–rHO-1, 10–30 nM rCPR—or DTT-treated 147CC514 or 10–100 nM 147CC514—and 25 µM NADPH in 0.1 M potassium phosphate buffer (pH 7.4). The reaction commenced upon the addition of NADPH. The initial rates of reduction of ferric heme–rHO-1 were calculated based on the decrease in the absorbance at 406 nm and the increase in the absorbance at 420 nm. The differences in ε between the ferric heme–rHO-1 and CO-bound ferrous heme–rHO-1 were 82.2 mM^−1^ cm^−1^ at 406 nm and 131 mM^−1^ cm^−1^ at 420 nm. A typical example is shown in Figure S2B. The mean values of the initial rates obtained were plotted against the concentration of rCPR. The rate constants for the reduction of heme in heme–rHO-1 were determined from the slope of the fitted line. The concentration of NADPH used in all assays was determined by measuring the absorbance at 340 nm (ε = 6.22 mM^−1^ cm^−1^). A Cary 50 Bio UV–visible spectrophotometer (Varian, Palo Alto, CA, USA) was used for spectroscopic measurements in the enzymatic assay.

### 2.3. Analytical Ultracentrifugation

Sedimentation equilibrium experiments were performed at 25 °C in 0.1 M potassium phosphate buffer (pH 7.4) in an Optima XL-A analytical ultracentrifuge (Beckman Coulter, Brea, CA, USA) with an An-60 Ti rotor. The data were acquired using the ProteomeLab XL-A software (Beckman Coulter). The mixtures of heme–rHO-1 and the equimolar CPR samples—rCPR, ΔTGEE, 147CC514, or IAM/DTT-treated 147CC514—were subjected to sedimentation equilibrium analysis. Aliquots of the protein solutions (120 μL) and reference buffer (140 μL) were loaded into a sedimentation equilibrium cell equipped with a double-sector charcoal–Epon centerpiece (12 mm path length). Following a 24 h equilibration period at 15,000 rpm, the absorbance was measured as a function of the radial position. The absorbance at 455 nm was measured in step mode with a step size of 0.001 cm and 18 replicates at each radial position. The solvent density (ρ) and partial specific volume (ῡ) were calculated using the SEDNTERP software (http://www.rasmb.bbri.org), and data analysis was performed using the Origin 9 software (OriginLab, Northampton, MA, USA). The sedimentation equilibrium data were fitted to a complex model, representing HO-1 + CPR ⇄ HO-1•CPR equilibrium (Equation (1)) [37].

A(r) = A_HO-1_(r_0_) exp { M_HO-1_ H_HO-1_ (r^2^ − r_0_^2^) } + A_CPR_(r_0_) exp { M_CPR_ H_CPR_ (r^2^ − r_0_^2^) } + *K*_HO-1•CPR_ A_HO-1_(r_0_) A_CPR_(r_0_) exp { (M_HO-1_ H_HO-1_ + M_CPR_ H_CPR_)(r^2^ − r_0_^2^) } + δ (1)

The data were also fitted to a non-complexed model in which two proteins exist in a monomeric state (Equation (2)).

A(r) = A_HO-1_(r_0_) exp { M_HO-1_ H_HO-1_ (r^2^ − r_0_^2^) } + A_CPR_(r_0_) exp { M_CPR_ H_CPR_ (r^2^ − r_0_^2^) } + δ (2)

In these equations, A(r) represents the total sample absorbance as a function of the radial position, r; A_HO-1_(r_0_) and A_CPR_(r_0_) represent the absorbances of monomeric heme–rHO-1 and CPRs at a reference position (r_0_), respectively; M_HO-1_ and M_CPR_ represent the average values of the molecular weights of heme–rHO-1 and CPRs, respectively; δ represents a minor baseline error correction term; H = (1−ῡρ)ω^2^/2RT, where ω represents the angular velocity of the rotor, R represents the gas constant, and T represents the absolute temperature; *K*_HO-1•CPR_ represents the association constant on the absorbance concentration scale, *K*_HO-1•CPR_ = A_HO-1•CPR_/A_HO-1_ A_CPR_. To determine the dissociation constant (*K*_d_), *K*_HO-1•CPR_ was converted to the molar scale as follows:

(3) $$K_{d}=\left(\frac{\varepsilon_{\mathrm{HO}-1}+\varepsilon_{\mathrm{CPR}}}{\varepsilon_{\mathrm{HO}-1}{\;\varepsilon }_{\mathrm{CPR}}\;l}\right)\;\left(\frac{1}{K_{\mathrm{HO}-1•\mathrm{CPR}}}\right)$$

where ε_HO-1_ and ε_CPR_ represent the molar extinction coefficients of heme–HO-1 and CPRs, respectively, and *l* represents the optical path length. The ε (mM^−1^ cm^−1^) values of heme–rHO-1, rCPR, ΔTGEE, 147CC514, and IAM/DTT-treated 147CC514 at 455 nm were 15.1, 24.0, 23.8, 23.7, and 23.8, respectively.

### 2.4. Cryo-Electron Microscopy (EM), Image Processing, and Molecular Modeling of the rCPR–Heme–rHO-1 Complex

Specimens of the rCPR–heme–rHO-1 complex were quick-frozen using liquid ethane and stored in liquid nitrogen. In brief, 5 μL of the heme–rHO-1 (2 μM, 0.06 mg/mL) and rCPR (1.4 μM, 0.10 mg/mL) mixture in 0.1 M potassium phosphate buffer (pH 7.4) containing 2 μM NADP^+^ was mounted on a holey carbon grid and blotted with filter paper to remove the excess liquid and create a thin aqueous layer. The grid was then plunged into an ethane slash at −185 °C to create a thin layer of vitreous ice. The prepared specimens were examined at normal liquid nitrogen temperature using a CT3500 cryo-holder (Oxford Instruments, Abingdon-on-Thames, UK) and was further subjected to EM. Cryo-electron micrographs were captured using an electron microscope (EF-2000, Hitachi, Tokyo, Japan) equipped with an Si2048-CFX (DALSA-MedOptics, Tucson, AZ, USA) CCD camera [38]. The EM conditions were as follows: acceleration voltage, 200 kV; defocusing values, 1 to 3 μm for contrast enhancement; direct magnification, 140 k. After contrast transfer function compensation, 513 particles (128 × 128 pixel/image) were interactively selected from the micrographs (Figure S3). Three-dimensional molecular modeling was performed using an extensible object-oriented system (Eos), as reported previously [39,40]. The overall resolution obtained for the reconstructed map of rCPR–heme–HO-1 was calculated as 25 Å by FSC_0.5_.

## 3. Results

### 3.1. Enzymatic Assay

**Figure 1:**
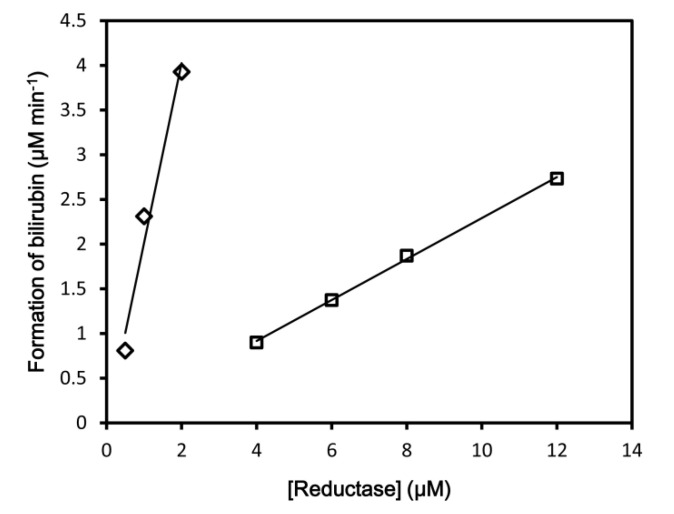
HO-1 activity. Bilirubin formation rates obtained in the NADPH–rCPR and NADPH–147CC514 systems were plotted as blank diamonds and squares, respectively. A representative example of the multiple measurements performed is displayed. See Methods for details.

First, we determined the enzymatic activities of rHO-1 in the NADPH–147CC514 system. The rate of bilirubin formation was 1.96 ± 0.35 min^−1^ (N = 3) in the NADPH–rCPR system, compared to 0.229 ± 0.0037 min^−1^ (N = 4) in the NADPH–147CC514 system (Figure 1, Table 1). Therefore, the activity was 8.6-fold lower in the 147CC514 system. This is consistent with previous assays that used cytochrome P450 2B4; cytochrome P450 activity in the NADPH–membrane-bound 147CC514 system was 9.3-fold lower than that in the NADPH–membrane-bound CPR system [27]. We also assessed rHO-1 activity in the presence of IAM/DTT-treated 147CC514, in which the disulfide bond between Cys147 and Cys514 was cleaved, and both cysteine residues were alkylated to prevent re-formation of the disulfide bond during the enzymatic assay. rHO-1 activity was similar in the NADPH–IAM/DTT-treated 147CC514 system and the NADPH–IAM/DTT-treated rCPR system, although the values were almost 60% of that obtained in the presence of non-treated rCPR (1.17 ± 0.0024 min^−1^ for IAM/DTT-treated rCPR and 1.25 ± 0.197 min^−1^ for IAM/DTT-treated 147CC514).

**Figure 2:**
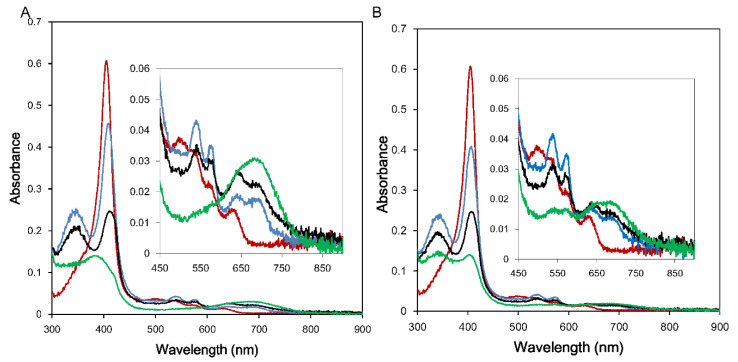
Changes in the absorption spectra of 4.3 μM heme–rHO-1 during the single turnover reaction in the presence of NADPH–rCPR or NADPH–147CC514. (**A**) Reaction with 0.04 μM rCPR. The spectra were recorded before (red) and 30 sec (cyan), 3.5 min (black), and 30 min (green) after NADPH addition. (**B**) Reaction with 0.1 μM 147CC514. The spectra were recorded before (red) and 3 min (cyan), 10 min (black), and 40 min (green) after NADPH addition. Magnified views of the visible region are in shown in the insets.

As stated above, the HO reaction is fairly complex and requires seven electrons from CPR. It is interesting to examine the action of 147CC514 in the HO reaction system. Therefore, we observed the single turnover HO reaction in the NADPH–147CC514 system. In the NADPH–rCPR (0.04 μM) system, the oxy-form was formed immediately, following which, the CO–verdoheme and the verdoheme forms appeared. Within 30 min, the heme residue was completely converted to biliverdin (Figure 2A). This appears to be similar to the reaction occurring in the NADPH–147CC514 (0.1 μM) system (Figure 2B).

### 3.2. Reduction Kinetics

**Figure 3:**
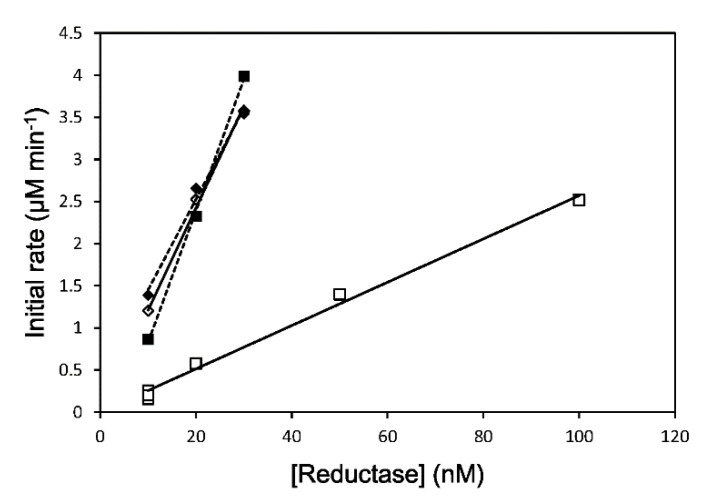
Rate of heme reduction in heme–rHO-1 in the NADPH–rCPR (diamonds) or NADPH–147CC514 systems (squares). The initial reduction rates of ferric heme–rHO-1 in the presence of rCPR or 147CC514 after the addition of NADPH were recorded under CO-saturated conditions. The results obtained using DTT-treated reductases are plotted as filled symbols and dashed lines. A representative example of the multiple measurements performed is displayed. See Methods for details.

The reduction rate of the ferric heme iron in heme–rHO-1 was measured by the rate of formation of CO-bound heme–rHO-1 in a CO-saturated atmosphere. The apparent reduction rate constants for the heme reduction of heme–rHO-1 were determined to be 122 ± 3.8 min^−1^ for rCPR (N = 3), 23.9 ± 5.1 min^−1^ for 147CC514 (N = 6), and 112 ± 23.6 min^−1^ for DTT-treated 147CC514 (N = 4) (Figure 3, Table 1) from the slope fitted by the least-square method. The rate of reduction in the presence of 147CC514 and DTT-treated 147CC514 was 5.1-fold and 1.1-fold slower, respectively, than that in the presence of rCPR. The data indicated that the decrease of the reduction rate was recovered by the cleavage of the disulfide bond between Cys147 and Cys514 in 147CC514. This is consistent with the results of the HO assay stated above and the rate of cytochrome *c* reduction in the presence of membrane-bound 147CC514 [27]. Previously, we reported that the rate of heme–rHO-1 reduction in the presence of ΔTGEE was 360-fold slower than that in presence of rCPR [24]. Therefore, 147CC514 was shown to be capable of reducing the ferric heme iron in heme–rHO-1 and, although its efficacy was observed to be limited, it was better than ΔTGEE.

### 3.3. Complex Formation between CPRs and Heme–rHO-1 Evaluated by Ultracentrifugation

**Figure 4:**
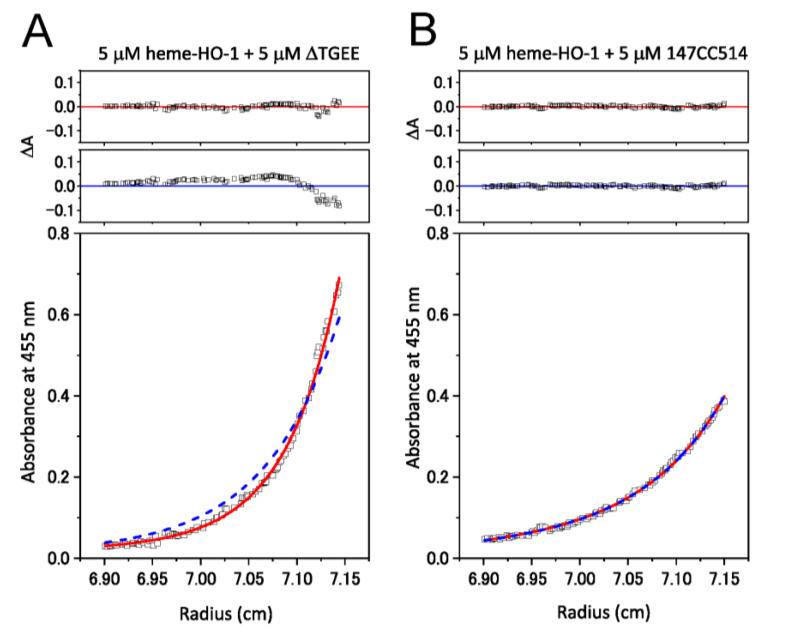
Sedimentation equilibrium analysis for heterodimer formation between heme–rHO-1 and ΔTGEE (**A**) or 147CC514 (**B**). The concentration of each protein was 5 μM. Absorbance data were collected at 15,000 rpm in a Beckman XL-A analytical ultracentrifuge. The data points fit to the complex (red lines) and non-complexed (blue dashed lines) models. The *K*_d_ value of ΔTGEE for heme–rHO-1 was estimated to be 0.178 ± 0.077 μM. Residual fitting is depicted above both curve fits, with the complex model indicated in red, and the non-complexed model in blue.

Sedimentation equilibrium experiments were performed to determine the affinity in the heterodimer formation between CPRs and heme–rHO-1. The sedimentation behaviors of heme–rHO-1 complexed with ΔTGEE or 147CC514 are compared in Figure 4. According to the data obtained for 5 μM heme–rHO-1 plus 5 μM ΔTGEE, a complex model fitted well with the data with small, symmetrically distributed residuals (Figure 4A, upper and bottom panels), whereas a non-complexed model fitted poorly with the data (Figure 4A, middle and bottom panels). The *K*_d_ value of ΔTGEE for heme–rHO-1 was estimated to be 0.178 ± 0.077 μM. Conversely, the data obtained from the reaction using 5 μM heme–rHO-1 plus 5 μM 147CC514 revealed a shift in the absorbance toward a smaller radial position, suggesting a shift in the equilibrium toward a significantly larger population of monomeric proteins (compare the lower panels of Figure 4A,B). When the concentration of heme–rHO-1 and 147CC514 increased to 15 μM (maximum concentration allowing the sedimentation equilibrium measurement), we recorded a *K*_d_ value of 111 ± 5 μM (Figure S4A). Notably, the *K*_d_ value of 147CC514 for heme–rHO-1 was reduced to 65.5 ± 17.0 μM upon the cleavage of the disulfide linkage between Cys147 and Cys514 and was comparable to that of rCPR (62.1 ± 1.7 μM) (compare Figure S4B,C).

### 3.4. Cryo-EM Structure of the rCPR–Heme–rHO-1 Complex

**Figure 5:**
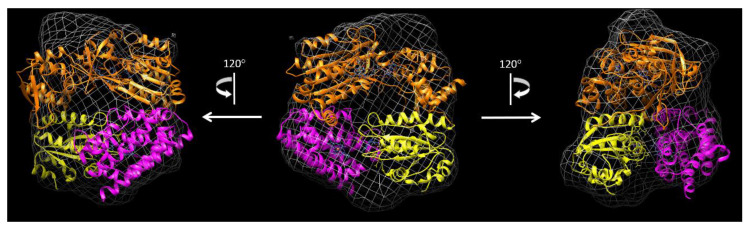
Three-dimensional reconstruction of the rCPR–heme–rHO-1 complex structure from a cryo-electron microscopic image (gray net); comparison with the X-ray structure of the ΔTGEE–heme–HO-1 complex (PDB ID: 3WKT). Resolution of the structure from cryo-EM was calculated as 25 Å. The X-ray crystal structures of the ΔTGEE–heme–HO-1 complex are indicated by ribbon diagrams: magenta, rHO-1; yellow, FMN domain of ΔTGEE; orange, FAD domain of ΔTGEE.

We could successfully observe single particles of the rCPR–heme–rHO-1 complex using cryo-EM. Figure 5 compares a cryo-electron microscopic image and the X-ray structure of the ΔTGEE–heme–HO-1 complex (PDB ID: 3WKT) [24]. The surface structure of rCPR–heme–rHO-1 (illustrated as a gray net in Figure 5) was consistent with the X-ray structure of the ΔTGEE–heme–HO-1 complex (ribbon diagrams in Figure 5), which confirmed that the X-ray structure represents the complex structure of rCPR–heme–HO-1 in aqueous solution.

## 4. Discussion

Several biophysical studies on CPR, including small-angle X-ray scattering, ion-mobility separation–mass spectroscopy, NMR, and fluorescence resonance energy transfer (FRET) studies, among others, suggest the presence of a redox-linked equilibrium between the open and the closed conformations of CPR [41,42,43,44]. The recently discovered crystal structure of the complex formed between ΔTGEE and heme–rHO-1 revealed that the open form of CPR binds tightly to heme–rHO-1 and the heme residue is positioned closed enough to FMN for electron transfer to occur from FMN to heme [24]. The cryo-EM map of the rCPR and heme–rHO-1 complex presented in this paper is consistent with the crystal structure, although the resolution of the EM map was lower (Figure 5). Further, we characterized HO activity, reduction kinetics, and affinity between the CPR variants and heme–rHO-1. We observed that, although heme–rHO-1 bound tightly to ΔTGEE (*K*_d_ = 0.178 ± 0.077 μM), the reduction kinetics in the presence of ΔTGEE were considerably slow [24]. The apparent affinity of heme–rHO-1 for 147CC514 (*K*_d_ = 111 ± 5 μM) was weaker than that for rCPR (*K*_d_ = 62.1 ± 1.7 μM). This is consistent with the results of HO activity and reduction kinetics. The HO activity and reduction kinetics in the presence of 147CC514 were 9-fold and 5-fold lower and slower, respectively, than those in the presence of rCPR (Table 1). The sedimentation equilibrium experiment suggested that the affinity between 147CC514 and heme–rHO-1 was restored upon cleavage of the unique disulfide linkage in 147CC514, and its *K*_d_ value (*K*_d_ = 65.5 ± 17.0 μM) was comparable to the apparent affinity between rCPR and heme–rHO-1 (Figure S4). The reduction of the disulfide bond in 147CC514 restored HO activity, reduction kinetics, and affinity of heme–rHO-1, which suggests that fixing the closed form by targeting the disulfide bond affects these characteristics.

However, 147CC514 retained the electron transfer activity to heme–rHO-1, although it was 10–20% of that of rCPR. The residual activity was consistent with the redox activity of 147CC514 with cytochrome *c* and cytochrome P450 [27]. We reasoned that a relatively small fraction of 147CC514 without the disulfide bond transfers electrons to heme–rHO-1. We crystallized 147CC514 and confirmed the formation of the disulfide bond. We observed that Cys514 has two alternative conformations: a major conformation (refined occupancy of 0.73) in which a disulfide bond is formed with Cys147, and a minor conformation (refined occupancy of 0.27) similar to that formed after DTT treatment (Figure S1) [27]. Because X-ray reduction during data collection might have cleaved the disulfide bond although the X-ray dose was limited to 0.4 MGy, this is consistent with the residual activity of 147CC514. Therefore, we believe that the closed form of CPR does not bind heme–HO-1; however, the closed form of CPR is necessary for intramolecular electron transfer. Our data suggest that both the closed and the open forms are necessary for efficient electron transfer from CPR to heme–HO-1.

Recent NMR and FRET studies suggest that the “open–close” transition in CPR has a time scale ranging from milliseconds to seconds [41,42], whereas that of the electron transfer may range from femtoseconds to nanoseconds [45,46]. Therefore, at least 10 milliseconds to 10 seconds are required to complete an HO reaction, which requires seven transition cycles. α-Verdoheme, one of the oxygen-labile intermediates of the HO reaction, would be protected by binding to CO, which is concomitantly released with α-verdoheme. The binding of CO to α-verdoheme is also related to the redox potential of α-verdoheme [47]. α-Hydroxyheme is more oxygen-labile than α-verdoheme, and it is considerably difficult to detect the intermediate by spectroscopy. Perhaps, there is a yet undiscovered mechanism that protects α-hydroxyheme in HO or accelerates electron transfer to α-hydroxyheme in HO.

## 5. Conclusions

We analyzed the interaction between heme–rHO-1 and an rCPR mutant fixed in a closed conformation, 147CC514, by analytical ultracentrifugation. Further, we assayed HO activity and heme reduction activity in the presence of the NADPH–147CC514 system. The results indicate that the interaction between heme–rHO-1 and 147CC514 is considerably weak relative to that between heme–rHO-1 and the rCPR mutant fixed in an open conformation, i.e., ΔTGEE. The enzymatic activity of 147CC514 is markedly weaker than that of rCPR. In addition, we confirmed that the crystal structure of ΔTGEE in complex with heme–rHO-1 is similar to the relatively low-resolution cryo-EM structure of rCPR complexed with heme–rHO-1 in solution. Thus, CPR in the open form could be tightly bound to heme–rHO-1 for intermolecular electron transfer, and CPR in the closed form is suitable for intramolecular electron transfer between coenzymes bound to CPR. We conclude that the “open–close” transition of CPR is indispensable for a rapid “association–dissociation” cycle and a smooth electron transfer from CPR to heme–HO-1.

## Figures and Tables

**Table 1:** Summary of the enzymatic assays. HO, heme oxygenase; rCPR, rat cytochrome P450 reductase; DTT, dithiothreitol; IAM, 2-iodoaceteamide.

Reduction System	HO Activity		Apparent Reduction Rate Constant	
	(min^−1^)	(%)	(min^−1^)	(%)
rCPR	1.96 ± 0.35	100	122 ± 3.8	100
147CC514	0.229 ± 0.0037	11.7 ± 0.19	23.9 ± 5.1	19.6 ± 4.2
DTT-treated rCPR	ND	ND	130 ± 10.8	107 ± 8.9
DTT-treated 147CC514	ND	ND	112 ± 23.6	91.8 ± 19
IAM/DTT-treated rCPR	1.17 ± 0.0024	59.7 ± 0.12	ND	ND
IAM/DTT-treated 147CC514	1.25 ± 0.197	63.8 ± 10	ND	ND
ΔTGEE [24]	ND	ND	0.250	0.205

## Supplementary Materials

The following are available online at https://www.mdpi.com/2076-3921/9/8/673/s1, Figure S1: (A) Close-up view of the disulfide bond in 147CC514 and (B) Comparison of our refined model with 3OJW. Figure S2: Raw timecourse data for the measurements of HO activity (A) and reduction rate (B). Figure S3: Cryo-EM data of rCPR–heme–rHO-1 complex. Figure S4: Sedimentation equilibrium analysis for heterodimer formation of heme–rHO-1 with 147CC514 (A), IAM/DTT-treated 147CC514 (B), and rCPR (C). Table S1: Crystallographic data collection and refinement statistics of 147CC514.
